# Case report of squamous cell cancer arising in perineal epidermal inclusion cyst, presenting as rapidly enlarging and cavitating lesion

**DOI:** 10.1016/j.ijscr.2018.10.054

**Published:** 2018-10-29

**Authors:** Jose Vale, Yijun Pang, Arthur Kumpf, David Fitkin, Scott Drew

**Affiliations:** a General Surgery, OhioHealth Marion Medical Campus, Marion Medical Campus, 1040 Delaware Avenue, 43302 Marion, OH, USA; b Anatomic and Clinical Pathology, OhioHealth Marion Area Physicians, Marion Medical Campus, 1040 Delaware Avenue, 43302 Marion, OH, USA; c Plastic and Reconstructive Surgery, OhioHealth Marion Area Physicians, Marion Medical Campus, 1040 Delaware Avenue, 43302 Marion, OH, USA; d Urology, OhioHealth Marion Area Physicians, Marion Medical Campus, 1040 Delaware Avenue, 43302 Marion, OH, USA; e Dermatology, OhioHealth Marion Area Physicians, Marion Medical Campus, 1040 Delaware Avenue, 43302 Marion, OH, USA

**Keywords:** Case report, Perineal, Epidermal inclusion cyst, Squamous cell cancer, Cavitating lesion

## Abstract

**Introduction:**

Cutaneous squamous cell cancer (cSCC) is a common condition, with straight forward and well-known heuristics regarding diagnosis, treatment, and surveillance. cSCC arising in an epidermal inclusion cyst is rare, but not unheard of. In contrast, the authors’ are unaware of any prior reports of cSCC arising in an epidermal inclusion cyst within an atypical cavernous and cavitating lesion in the perineum.

**Presentation of case:**

A 48-year-old male presented with a cavitating and rapidly growing cutaneous perineal lesion. Preliminary diagnostic procedures (biopsy) showed benign pathology and imaging showed no involvement of perineal organs such as prostate or rectum. Discordance between initial diagnostics and clinical features prompted multi-specialty consultation, including dermatological pathology. Repeat biopsy yielded in situ, possibly invasive squamous cell cancer, arising in the background of an epidermal inclusion cyst. Wide excision with advancement flap reconstruction was employed, with temporary diverting colostomy to avoid perineal sepsis.

**Discussion:**

Location and appearance of this lesion provided a diagnostic and treatment challenge requiring multi-specialty involvement for diagnosis and successful treatment. Specialists from urology, general surgery, dermatology, general and dermatological pathology, plastic and reconstructive surgery, colorectal surgery and medical and radiation oncology were involved in diagnosis, treatment plan and execution. None of the specialists involved had seen this singular presentation before, and this created an ownership and management challenge.

**Conclusion:**

The cavitating, atypical appearance of lesion complicated diagnosis. Location required pre-treatment coordination and deliberation between dermatology, urology, general surgery, and plastic surgery. Malignant degeneration of inclusion cysts, although rare, has to be kept in mind, especially when presenting with rapid growth, and persistence in ruling out malignant diagnosis is fundamental.

## Highlights

• Perineal inclusion cysts may harbor clinically occult malignant degeneration.
• Histological analysis of large inclusion cysts required to rule out occult malignancy.
• Perineal cysts with malignant degeneration are diagnostic, reconstructive challenge.
• Risk of malignancy in perineal inclusion cysts helps treatment decisions.
• Large perineal cyst requires multidisciplinary surgical team for excision and closure.
• Colostomy as adjunct of perineal reconstruction after excision of inclusion cyst.
• Perineal inclusion cysts may present as atypical, cavitating lesions.

## 1 Introduction

This work has been reported in line with the SCARE criteria [1].

Epidermal inclusion cysts and squamous cell carcinoma of skin are common lesions. Squamous cell carcinoma arising in a perineal epidermal inclusion cyst is rare, this is the 5^th^ case in literature up to date [2]. This may be the only report of a squamous cell carcinoma arising in a cavitating, epidermal inclusion cyst of the perineum.

## 2 Presentation of case

**Fig. 1:**
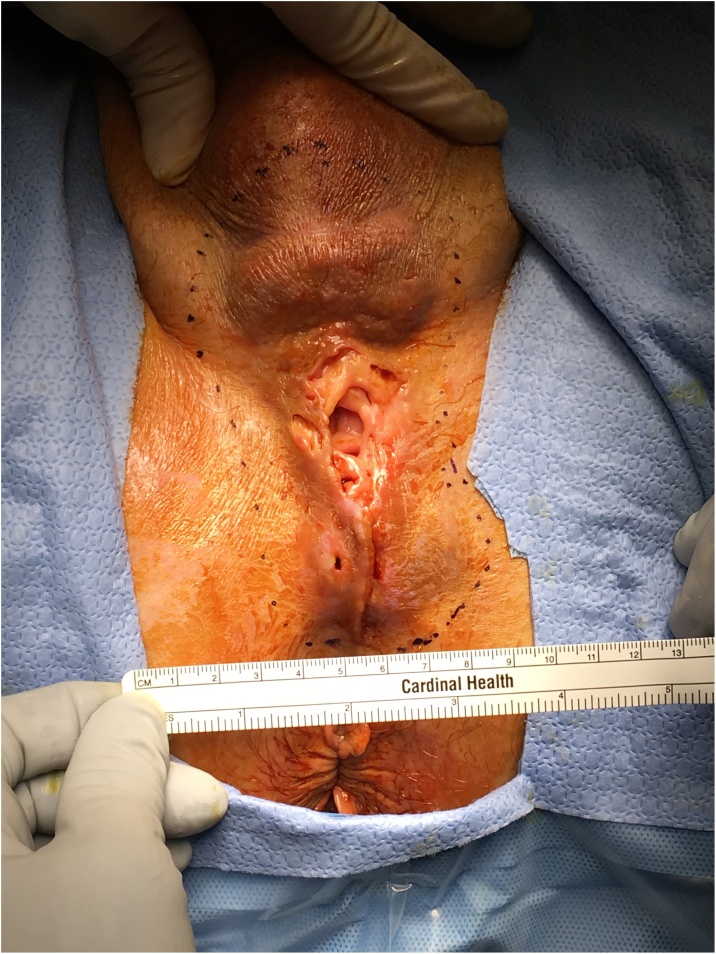
Cavitating perineal inclusion cyst, pre excision.

This case was managed in a community hospital in the Midwest within a multi-hospital, not-for-profit system. A 48-year-old male patient presented to his primary care physician complaining of a tender perineal mass of about one month of evolution. Patient has history of metabolic syndrome defined by type 2 diabetes mellitus, obesity, coronary artery disease, ischemic cardiomyopathy, congestive heart failure and hypertension. He is a social drinker and smokes about a pack of cigarettes per day. Physical examination showed a rubbery, tender and cavitating lesion in perineum, in midline, about equidistant from scrotum and anal verge. This lesion measured about 5 cm in diameter, with associated underlying soft tissue induration, extending from scrotum anteriorly to about one centimeter from anal verge posteriorly. (Fig. 1) The cavitating portion was moist and irritated, and patient complained of discomfort to touch. Upon examination by his primary physician, the atypical appearance of perineal lesion prompted urgent urological consultation.

**Fig. 2:**
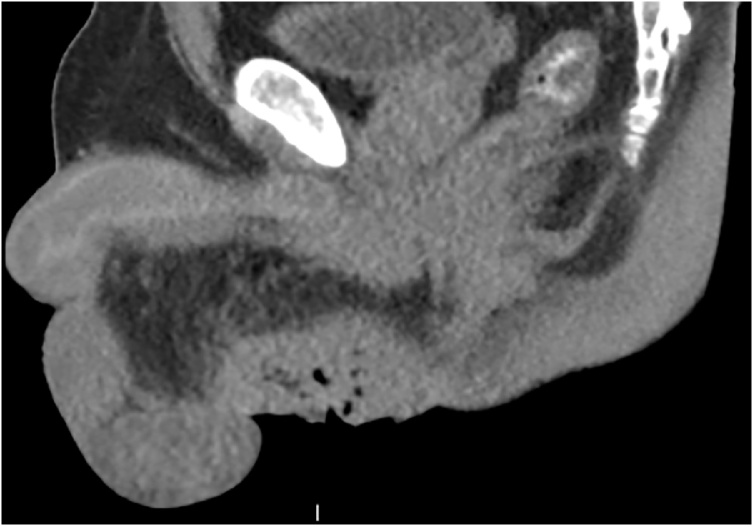
Pre op CT scan.

Patient was seen later that morning by urology who in turn obtained immediate evaluation by general surgery. No clear cut etiology could be determined by physical examination by either specialist; therefore, the decision was made to perform abdomen pelvic CT scan and biopsy. CT scan showed a cavitating cutaneous lesion, without any involvement of anorectal or urological structures (Fig. 2). Lesion biopsy - performed under anesthesia - indicated a benign cystic papilloma.

The benign nature of initial histological diagnosis was discordant with the patient’s history of rapid growth, which suggested a malignant etiology. Therefore, biopsy under anesthesia was repeated, with wider and more aggressive sampling of the lesion. Histological analysis once again showed benign results: keratinizing squamous epithelium with inflammation and fibrosis.

**Fig. 3:**
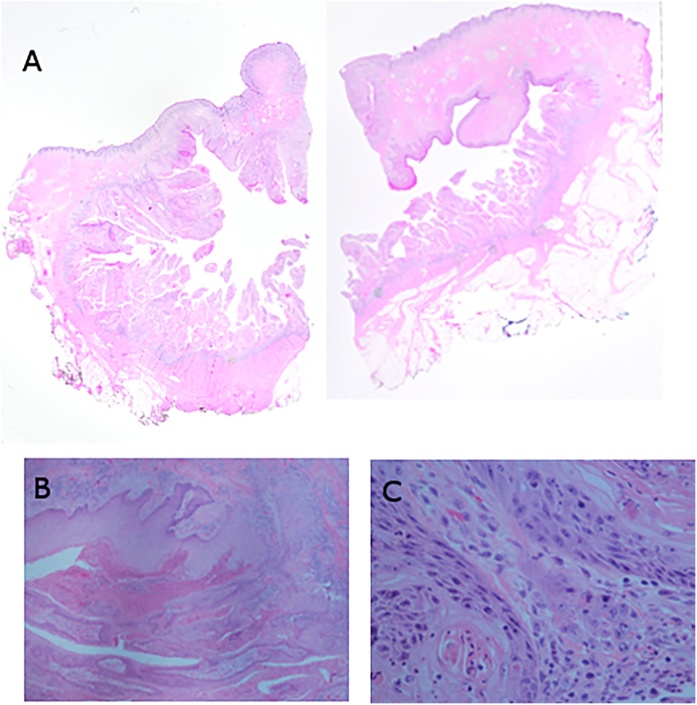
Post excision histology.

Despite consecutive benign biopsy results, we were convinced this lesion had to be malignant. Given the risks of prolonged disability or permanent mutilating complications associated with oncological excision of skin in this anatomical location, we felt that obtaining accurate preoperative diagnosis was fundamental for a sound informed consent from patient. We therefore requested evaluation and second opinions from dermatology and dermatological pathology. A repeat biopsy yielded in situ, and possible invasive squamous cell cancer, arising in the background of an epidermal inclusion cyst, probably related to HPV infection (Fig. 3a–c).

The case was presented to Tumor Board and an independent evaluation by colorectal surgery in tertiary medical center was obtained. The summary of the multi-specialty panel was to treat this cutaneous lesion by wide excision. Radiotherapy was discussed as a non-surgical management. Radiation oncology opined that radiation would only be indicated in the setting of positive margins where re-excision would be destructive or permanently disabling to patient. Given the size of the lesion, closure of wound would require either a skin graft or advancement flap, a decision to be made by plastic and reconstructive surgery at time of operation. To avoid perineal sepsis and improve odds of successful wound healing, it was decided to perform a temporary diverting colostomy.

**Fig. 4:**
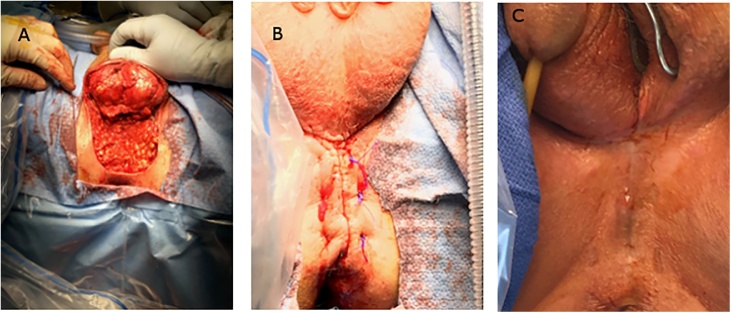
A: post wide excision, B: s/p closure by flap advancement, C: perineum 6 months post excision during takedown of colostomy.

Patient received preoperative evaluation by cardiology and preoperative risk factors were optimized. Following laparoscopic end colostomy and Hartmann pouch as the first step in surgery, wide excision of the perineal lesion was performed by a urology and general surgery team (Fig. 4a). Reconstruction was performed by advancement flaps; elasticity of the scrotum facilitated closure (Fig. 4b). The patient recovered uneventfully, with only slight dehiscence of wound closure in central portion of repair. Margins of the resection were negative and no adjuvant treatment was advised by medical and radiation oncology. Once healing was complete at approximately 6 months post-surgery (Fig. 4c) patient was taken back to operating room to undergo robotic assisted laparoscopic closure of colostomy. He recovered well from that intervention. An incisional hernia in colostomy closure site was repaired 6 months after closure of colostomy.

## 3 Discussion

This case presented a diagnostic and treatment challenge. It was a cutaneous malignancy located in an anatomical location that proved to be a no man’s land for all specialties involved. None of the specialist involved had seen a case like this before. Perineal inclusion cysts are rare, and malignant degeneration even more so, with only 4 cases reported previous to this case.(2) Every involved specialty was essential to final outcome, yet no single specialty was qualified to provide diagnosis and treatment by itself. Successful management required pre-treatment coordination and deliberation, to work around the ownership problem. Dermatology provided definitive diagnosis. Urology provided expertise as to scrotal skin excision. General surgery provided diverting colostomy and perineal/perianal excision. Plastic surgery provided wound closure via advancement flap. Advancement flap closure was significantly enhanced by the elasticity of the scrotum. We believe that avoiding a skin graft facilitated greatly this operation and patient’s recovery.

The etiology of the squamous cell carcinoma is not certain. Possible Human Papilloma Virus (HPV) infection was investigated with in situ hybridization; however, no high risk HPV was identified.

## 4 Conclusion

Squamous cell cancer of skin is a common diagnosis. Its presence in an epidermal inclusion cyst, is not. There are a handful of case reports of malignant degeneration of inclusion cysts, most of them occurring in head and neck [3].There are 4 previous reports of squamous cell degeneration in a perineal inclusion cyst, to the date of this report. The gross appearance of the lesions in those reports is that of a cystic lesion containing sebum and keratin. Some of these lesions can reach large size, but their cystic nature and sebum/keratin content makes diagnosis straightforward [4]. The cavitating appearance of this lesion is singular, and none of the authors are aware of a previous report of a lesion of this appearance. This keratinizing lesion was open and no accumulation of sebum was present, which confused diagnosis. This lesion presented as a rapidly enlarging cavity in the central location of perineal skin, composed of keratinizing squamous epithelium. Clinical behavior was discordant with initial benign histological diagnosis. This feature underlies the fact that although uncommon, malignancy has to be kept in mind in lesions of this nature.

The location of this lesion, in the central portion of perineal skin, presented a treatment challenge requiring a multidisciplinary team both for diagnosis and for definitive treatment. Neither urology, colorectal or general surgery felt in complete ownership of the condition, unlike cystic lesions that are clearly located in genitalia or anorectal structures [5,6]. The location of malignant degeneration, deep within the cavitating portion of the lesion provided a diagnostic challenge. Behavior of lesion was very suspicious for malignancy, but without histological confirmation a priori, we were hesitant to proceed with a potentially morbid or disabling resection. Confirmation of malignant diagnosis simplified pre op peer review process and provided for a meaningful informed consent from patient.

Given the potentially catastrophic nature of possible complications, a temporary colostomy was placed pre-operatively. This decision may be challenged in future similar cases. We believe it was the right decision, given the patient’s characteristics. An accurate pre-operative diagnosis made treatment decisions easier on the treatment team, and facilitated patient’s acceptance of treatment options, especially the temporary colostomy. We hope that the heuristics used in management of this case will help guide other multidisciplinary teams in future decision making when faced with similar pathology and anatomical location.

## Conflicts of interests

The authors declare no conflicts of interests or competing interests.

## Funding

There were no sources of funding.

## Ethical approval

I declare that ethical approval has been exempted by my institution for this case report by the OhioHealth Office of Human Subjects Protections.

## Consent

Written informed consent was obtained from the patient for publication of this case report and accompanying images. A copy of the written consent is available for review by the Editor-in-Chief of this journal on request.

## Author contribution

Jose Vale MD: wrote paper, collected pictures, assembled treatment team, principal attending in care of patient.

Yujing Pang MD: pathological analysis, provided pictures of pathology specimen.

Arthur Kumpf MD: plastic and reconstructive surgeon in charge of wound closure.

David Fitkin MD: urologist, received initial referral, assisted during surgery in scrotal excision.

Scott Drew DO: provided second opinion and obtained specimen that proved malignancy in lesion.

## Registration of research studies

Not applicable.

## Guarantor

Jose Vale.

## Provenance and peer review

Not commissioned, externally peer reviewed.

## Declarations of interest

None.
